# Designing Novel Compound Candidates Against SARS-CoV-2 Using Generative Deep Neural Networks and Cheminformatics

**DOI:** 10.3390/ijms262412017

**Published:** 2025-12-13

**Authors:** Shang-Yang Li, Chin-Mao Hung, Hsin-Yi Hung, Chih-Wei Lai, Meng-Chang Lee

**Affiliations:** 1 Graduate Institute of Public Health, College of Public Health, National Defense Medical University, Taipei City 114201, Taiwan; young19990726@gmail.com; 2 Institute of Preventive Medicine, National Defense Medical University, New Taipei City 237010, Taiwan; chinmaohung@yahoo.com.tw; 3 Graduate Institute of Medical Sciences, College of Medicine, National Defense Medical University, Taipei City 114201, Taiwan; 4 School of Pharmacy, College of Medicine, National Cheng Kung University, Tainan 70101, Taiwan; z10308005@email.ncku.edu.tw; 5 College of Pharmacy, National Defense Medical University, Taipei City 114201, Taiwan; penghupharmacy@mail.ndmutsgh.edu.tw

**Keywords:** generative deep neural networks, reinforcement learning, cheminformatics, drug design, SARS-CoV-2, molnupiravir

## Abstract

The COVID-19 outbreak has had a tremendous socioeconomic impact around the world, and although there are currently some drugs that have been granted authorization by the U.S. FDA for the treatment of COVID-19, there are still some restrictions on their use. As a result, it is still necessary to urgently carry out related drug development research. Deep generative models and cheminformatics were used in this study to design and screen novel candidates for potential anti-SARS-CoV-2 small molecule compounds. In this study, the small molecule structure of Molnupiravir which has been authorized by the U.S. FDA for emergency use was used to be a model in a similarity search based on the BIOVIA Available Chemicals Directory (BIOVIA ACD) database using the BIOVIA Discovery Studio (DS) software (version 2022). There were 61,480 similar structures of Molnupiravir, which were used as training dataset for the deep generative model, and then the reinforcement learning model was used to generate 6000 small molecule structures. To further confirm whether those molecule structures potentially possess the ability of anti-SARS-CoV-2, cheminformatics techniques were used to assess 38 small molecule compounds with potential anti-SARS-CoV-2 activity. The suitability of 38 small molecule structures was calculated using ADMET analysis. Finally, one compound structure, Molecule_36, passed ADMET and was unpatented. This study demonstrates that Molecule_36 may have better potential than Molnupiravir does in affinity with SARS-CoV-2 RdRp and ADMET. We provide a combination of generative deep neural networks and cheminformatics for developing new anti-SARS-CoV-2 compounds. However, additional chemical refinement and experimental validation will be required to determine its stability, mechanism of action, and antiviral efficacy.

## 1. Introduction

SARS-CoV-2 (Severe Acute Respiratory Syndrome Coronavirus 2) is the virus that caused the COVID-19 pandemic after a pneumonia cluster occurred in Wuhan, China, in December 2019 [1]. By March 2023, 670 million people had been diagnosed, and over 6 million died, with a fatality rate of 1.02 [2].

However, given the lack of a promising therapeutic approach for COVID-19, more research about the development of drug candidates is needed [3]. Although there are currently some drugs that have been used for the treatment of COVID-19, there are some restrictions on their use [4,5]. As a result, other oral drugs for the treatment of COVID-19 are urgently needed to relieve the spread of the pandemic.

Conventional drug development is a long, costly, and arduous process [6]. Many external factors may cause promising candidates to be eliminated during the process; accordingly, only about 5 of the 5000 drug candidates will advance to clinical trials, and only about 1 will be approved for marketing [7]. Artificial intelligence-based drug development is regarded as an effective tool in reducing the time and cost required for conventional drug development [8]. For example, in target identification, artificial intelligence (AI) can be used to integrate databases to easily understand the relationship between disease and drug activity or to efficiently process large amounts of chemical data in order to further optimize absorption, distribution, metabolism, excretion, and toxicity (ADMET) profiles [9]. Currently, the majority of drug development research is carried out using the principles and techniques of Machine Learning (ML) in the field of AI [10], among which the most common methods are reinforcement learning (RL), supervised learning, and unsupervised learning [11]. AI techniques may effectively and quickly exclude drugs that are not potentially active, improving the efficiency of drug development and reducing the time and cost required [12]. Particularly, deep neural networks (DNNs) have increasingly supported medicinal chemistry across early discovery, from de novo molecular generation to property optimization. Generative models, for example, variational autoencoders (VAE), GANs (Generative Adversarial Network), and RL frameworks, have been used to design drug-like small molecules under synthesizability and drug-likeness constraints [13,14,15]. Gómez-Bombarelli et al. introduced the classic VAE framework for molecular generation/optimization [13]. De Cao and Kipf’s MolGAN shows GAN-based molecular graph generation [14]. Olivecrona et al. demonstrated deep RL for molecular de novo design with property constraints [15].

In September 2019, Insilico Medicine released a deep generative model (Generative tensorial reinforcement learning, GENTRL), a deep learning method for performing de novo drug design [16]. Zhavoronkov et al. identified a novel potent inhibitor of discoidin domain receptor 1 (DDR1) in 21 days using GENTRL. The study took only 46 days from screening to synthesis and cost a tremendous fraction of the cost of conventional drug development. The novel compound designed by Zhavoronkov has now completed the Phase I clinical trial, the first AI-based inhibitor to be approved. In addition, generative deep learning has been used to rapidly propose novel SARS-CoV-2 inhibitor candidates, with Zhavoronkov et al. targeting the viral 3CLpro early in the COVID-19 pandemic [17]. More recently, Transformer-based de novo design frameworks have been employed to generate small antiviral molecules directed against the SARS-CoV-2 RNA-dependent RNA polymerase (RdRp) [18]. Parallel advances in AI-assisted drug-target interaction modeling further enable rapid triage of large chemical spaces by coupling learned molecular representations with protein sequence/structure features. These directions collectively motivate integrating deep generative modeling with downstream silico screening to prioritize tractable candidates. The development of deep generative models for de novo drug design is of specific interest for the generation of novel small molecule compounds with drug properties that can bind to protein targets, making them well suited for the urgent SARS-CoV-2 drug development research [19].

Cheminformatics methods are essential for narrowing AI-generated chemical space into experimentally testable leads, as demonstrated in numerous studies on SARS-CoV-2 antiviral discovery [18,19,20,21,22,23]. For example, de novo designed molecules have been prioritized through integrated cheminformatics workflows combining pharmacophore matching [24], structure-based molecular docking to viral targets [19,21], binding free-energy estimation or scoring [21,22], and early ADMET and drug-likeness filtering to yield synthesis-ready candidates [24]. These examples highlight how cheminformatics can efficiently down-select AI-generated candidates by evaluating their target engagement, physicochemical properties, and medicinal chemistry feasibility before synthesis. Pharmacophore-guided filtering, structure-based docking, and drug-property screening have been widely incorporated into drug design pipelines to refine computationally generated molecules into feasible antiviral leads [25].

Among AI methods used in drug discovery (e.g., predictive drug-target interactions models, similarity search), generative deep neural networks (VAEs, GANs, and RL-augmented frameworks) are uniquely suited to create novel, drug-like molecules beyond enumerated libraries while optimizing multiple objectives (activity, ADMET, and synthesizability) in a single workflow [19,20]. Prior studies demonstrated that generative models can rapidly deliver synthesis-ready chemotypes: GENTRL produced potent DDR1 inhibitors on an accelerated timeline [13]; GAN/RL-based de novo design generated candidate inhibitors for SARS-CoV-2 3CLpro early in the pandemic [17]; and Transformer-based generators have been applied to antiviral/RdRp-oriented design [18]. These successes, together with the ease of coupling generative outputs to pharmacophore-guided filtering and structure-based docking, motivated our selection of a modified GENTRL (mGENTRL) as the generative core of our pipeline in this study.

## 2. Results

### 2.1. Generation of New Chemical Structures

**Figure 1:**
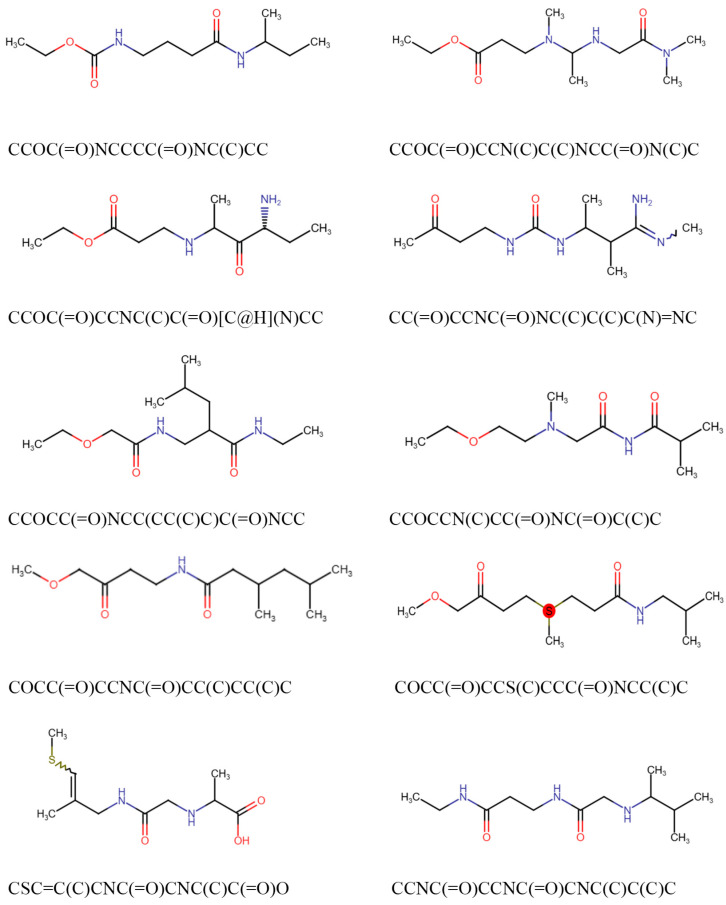
Examples of newly generated compounds by mGENTRL model. Some examples of mGENTRL model products.

The normal structural chemical formula of Molnupiravir was shown in Supplementary Figure S1A. We used DS software to predict any resonance structures of Molnupiravir, and two resonance structures were generated, of which the lower energy structure was chosen for further analysis (Supplementary Figure S1B). For generation of Molnupiravir-like structures in the training dataset of the mGENTRL model, a ligand-based similarity search of the preprocessed Molnupiravir resonance structure was conducted using the ACD database. A total of 61,480 compounds with similar structures to Molnupiravir were generated (Supplementary Figure S2). Next, the database of similar structures was fed into mGENTRL model, and the reinforcement learning mechanism was used to recognize and generate chemical structures matching the reward function. To validate whether this model produced valid SMILES, we examined the percentages of valid SMILES products and obtained a highly successful average rate at 98.87% of valid SMILES, suggesting its ability to produce valid SMILES. Finally, this model sampled 6000 chemical structures, examples shown in Figure 1.

### 2.2. Pharmacophore-Based Virtual Screening and Molecular Docking

**Figure 2:**
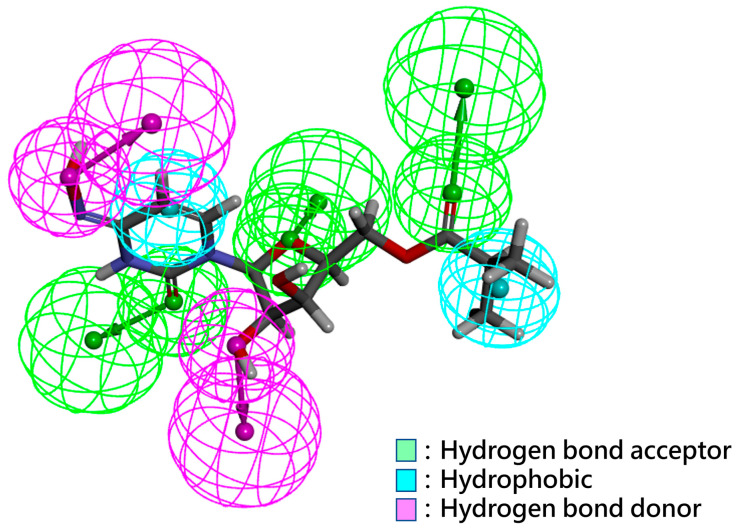
A pharmacophore model based on Molnupiravir was generated using DS software. The chemical properties of Molnupiravir were identified as shown in the Hydrogen bond acceptor, Hydrophobic, and Hydrogen bond donor.

A pharmacophore model based on Molnupiravir was created using DS software.Two hydrogen-bonded donors, three hydrogen-bonded acceptors, and two hydrophobic chemical features were identified as shown in Figure 2. Based on this pharmacophore model, 6000 chemical structures from mGENTRL were matched with pharmacophore features. There were 277 chemical structures with similar chemical properties to Molnupiravir identified. Subsequently, 277 chemical structures were docked to SARS-CoV-2 RdRp to identify potential candidate small molecules with a binding affinity greater than that of Molnupiravir. The protein used for molecular docking in this study was preprocessed, and the active site was configured as shown in Supplementary Figure S3, which contained the major amino acids that have been identified as potent inhibitors of SARS-CoV-2 RdRp, such as GLY616, TRP617, ASP618, TYR619, PRO620, LYS621, CYS622, LEU758, SER758, and CYS622. LEU758, SER759, ASP760, ASP761, ALA762, ALA797, LYS798, CYS799, TRP800, HIS810, GLU811, PHE812, CYS813, SER814, and GLN815, etc. Furthermore, ASP760, ASP761, LYS798, and SER814 were noticed to be essential amino acids for stabilizing the core structure of RdRp in relevant studies [26].

Subsequently, Molnupiravir and 277 chemical structures were docked to the active site of 7OZV. There were 38 chemical structures with better binding affinity than that of Molnupiravir (Supplementary Table S1).

### 2.3. Prediction of Properties of Blood–Brain Barrier and Intestinal Absorption Using DS

**Figure 3:**
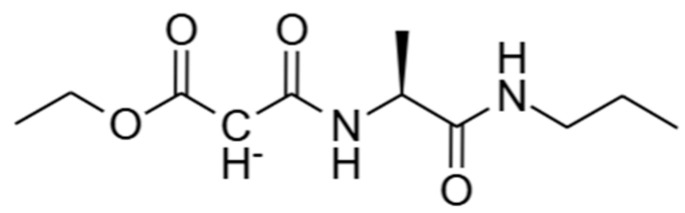
The 2D structural chemical formula of Molecule_36.

As shown in Supplementary Table S1, 38 small molecules screened by molecular docking simulation were analyzed to predict human intestinal absorption and BBB permeability using DS. Finally, Molecule_36 (the IUPAC name: (S)-1-ethoxy-1,3-dioxo-3-((1-oxo-1-(propylamino)propan-2-yl)amino)propan-2-ide), a small molecule compound with potential drug properties, was predicted to have good human intestinal absorption as well as low BBB permeability. The 2D structural chemical formula of Molecule_36 was shown in Figure 3.

### 2.4. Prediction of Molecule_36 Target Amino Acids of RdRp and Comparison with That of Molnupiravir

**Figure 4:**
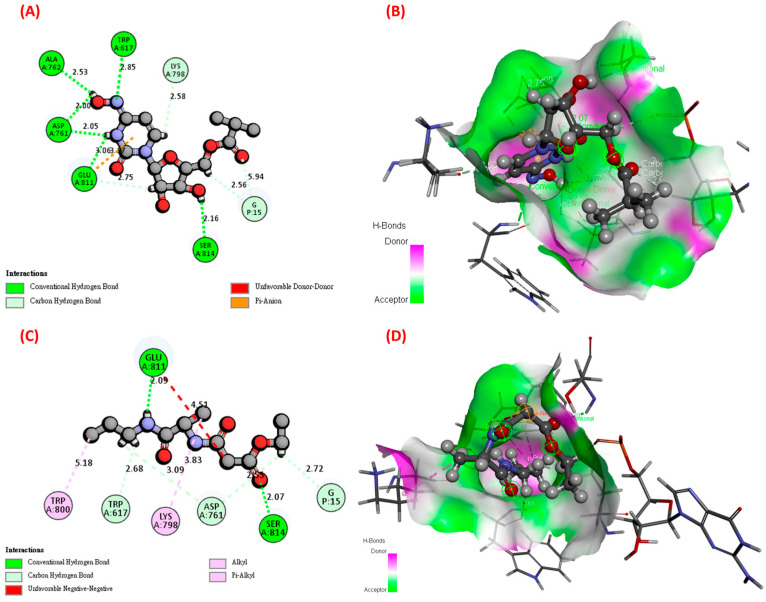
Molecular docking simulation of Molnupiravir and Molecule_36 with SARS-CoV-2 RdRp. (**A**) 2D interaction plot of Molnupiravir with SARS-CoV-2 RdRp. (**B**) Binding orientation of Molnupiravir at the active site of SARS-CoV-2 RdRp. (**C**) 2D interaction plot of Molecule_36 with SARS-CoV-2 RdRp. (**D**) Binding orientation of Molecule_36 at the active site of SARS-CoV-2 RdRp.

To further confirm which amino acids of RdRp were the targets of Molecule_36 and compare whether these targets were similar or differential from that of Molnupiravir, we conducted extra molecular docking simulations. Figure 4 depicted the molecular docking simulation results of Molnupiravir and Molecule_36. Molnupiravir formed four types of interaction bonds, including a Conventional Hydrogen Bond (TRP617, ASP761, ALA762, GLU811, and SER814), Carbon Hydrogen Bond (LYS798 and G15), Pi-Anion, and Unfavorable Donor–Donor (ALA762). Molecule_36 contained five types of interaction bonds, including Conventional Hydrogen Bonds (GLU811 and SER814), Carbon Hydrogen Bonds (TRP617, ASP761, and G15), Pi-Alkyl (TRP800), Alkyl (LYS798), and Unfavorable Negative–Negative (GLU811). There were several common points between Molnupiravir and Molecule_36, including Conventional Hydrogen Bond (GLU811 and SER814) and Carbon Hydrogen Bond (G15). Furthermore, Molecule_36 also formed interaction bonds with target amino acids (ASP761, LYS798, and SER814) of SARS-CoV-2 RdRp, suggesting its potential to inhibit SARS-CoV-2.

### 2.5. Comprehensive Prediction of ADMET Property of Molecule_36 and Molnupiravir Using Multiple Resources

Finally, tools, SwissADME, pkCSM, and DS software, were used to predict, analyze, and compare the ADMET properties of Molecule_36 and Molnupiravir shown in Table 1 [27]. Molecule_36 and Molnupiravir were both predicted to have good water solubility [28], and Molecule_36 had good intestinal absorption in humans [29]. Furthermore, Molecule_36 had a high Caco-2 permeability, implying that it had good drug absorption [30]. Both Molecule_36 and Molnupiravir were predicted to be non-P-glycoprotein (P-gp) substrates, implying that they would not be excluded by P-gp and could achieve drug absorption and bioavailability [31]. Bioavailability scores for Molecule_36 and Molnupiravir were similar [32].

Molecule_36 had a low steady-state volume of distribution (VDss) due to intracorporeal distribution properties, implying that its distribution in vivo was primarily into plasma rather than tissues [33]. Furthermore, because the BBB and the central nervous system were less permeable [34], it was speculated that Molecule_36 was unable to penetrate the BBB and enter the central nervous system. According to multiple tools predictions, Molecule_36 and Molnupiravir were not inhibitors of cytochrome P450 metabolizing enzymes (CYP1A2/CYP2C19/CYP2C9/CYP2D6/CYP3A4) [35,36]. Therefore, it was speculated that the possibility of adverse drug reactions and interactions was low [37]. Total clearance was the ratio of small molecule clearance in the liver and kidney. The total clearance of Molecule_36 was predicted to be greater than that of Molnupiravir, so it was assumed that Molecule_36 was superior to Molnupiravir due to its characteristic of being excreted from the body [38]. However, neither Molecule_36 nor Molnupiravir was a substrate for renal organic cation transporters 2 (OCT2), so it may not be possible to eliminate them by transporting them to the kidney via OCT2 [39].

Molecule_36, as predicted by DS, may not be toxic, whereas Molnupiravir was. The LD50 was the acute toxicity level of a chemical [40] and was predicted to be 6.58 g/kg for Molecule_36 and 3.03 g/kg for Molnupiravir, indicating that Molecule_36 was less toxic than Molnupiravir. Furthermore, neither was predicted to be carcinogenic or mutagenic. The pkCSM predicted that Molecule_36 and Molnupiravir had no potential to inhibit hERG (human ether-a-go-go gene). One of the causes of long QT syndrome was hERG-coded potassium channel inhibition, which may increase the risk of arrhythmias [41]. Moreover, Molecule_36 was predicted to not be skin sensitizing nor to exhibit hepatotoxicity.

SwissADME predicted a Synthetic Accessibility (SA) score of 2.5 for Molecule_36 and 4.49 for Molnupiravir. The SA score ranges from 1 to 10, with lower scores indicating simpler synthesis [42]. According to PubChem, Molecule_36 is a novel and unpatented small molecule compound. As a result, we conclude that Molecule_36 may be a potential novel anti-SARS-CoV-2 RdRp small molecule compound. Detailed data are shown in Supplementary Tables S2–S5.

### 2.6. Analysis of Molecule_36 Molecular Dynamics Simulations

**Figure 5:**
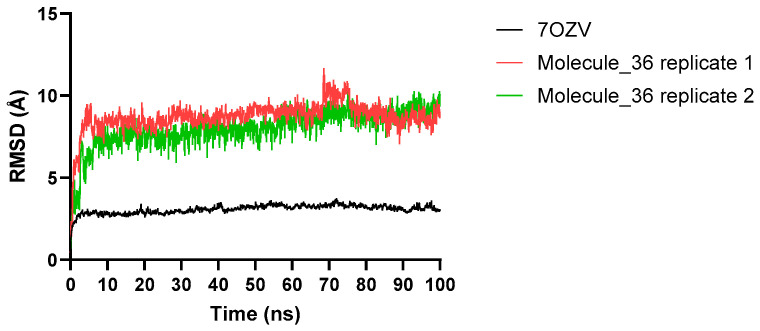
The root mean square deviation of 7OZV (RdRp) in complexes with Molecule_36.

RMSD (Root mean square deviation) trajectories were calculated for the RdRp-Molecule_36 complexes throughout 100 ns NAMD (Nanoscale Molecular Dynamics) production runs. For the reference RdRp structure (7OZV, black trace), the RMSD increased during the initial equilibration phase and reached ~2–3 Å within the first 5 ns. Thereafter, the trajectory remained stable, fluctuating narrowly around 3 Å for the remainder of the 100 ns simulation, consistent with an equilibrated polymerase core as reported in previous molecular dynamics (MD) simulations of SARS-CoV-2 RdRp complexes [43,44] (Figure 5). For the RdRp-Molecule_36 complexes, the RMSD rose rapidly during the first 5 ns to approximately 7–8 Å, reflecting early relaxation and adjustment of the protein–ligand complexes (Figure 5). From ~10 ns onward, the RMSD fluctuated within a relatively stable window of ~7–9 Å, with only a transient increase around 60–70 ns that subsequently relaxed back to the same range. Overall, the RMSD showed no persistent drift or structural disruption, suggesting that the complexes remained stable over the 100 ns simulation.

Results of two independent molecular dynamics simulations of Molecule_36 were shown.

## 3. Discussion

### 3.1. Comparative Interaction Analysis of Molnupiravir and Molecule_36

In this study, a workflow integrating a modified deep generative model (mGENTRL) and cheminformatics analysis was applied to identify potential SARS-CoV-2 RdRp inhibitors using Molnupiravir as a reference structure. Through ligand-based similarity search, deep generative modeling, pharmacophore-guided virtual screening, and structure-based docking, Molecule_36 was prioritized as a tractable lead with encouraging pharmacological and safety profiles. RdRp is indispensable for SARS-CoV-2 RNA replication and transcription [45]. Its lack of human homologs makes it a highly selective antiviral target, allowing the design of inhibitors with strong activity and minimal off-target effects [46]. Comparative docking analysis indicated that Molecule_36 interacts with catalytically important residues of RdRp, including TRP617, ASP761, LYS798, GLU811, and SER814, which overlap with the binding sites reported for Remdesivir and Molnupiravir [16]. Specifically, both Molnupiravir and Molecule_36 shared key binding features, including a Conventional Hydrogen Bond (GLU811 and SER814) and Carbon Hydrogen Bond (G15). In Ahmad’s docking analyses, the guanosine moiety of the tested nucleoside/ligand was reported to form hydrogen bonds with GLU811 and SER814, as well as with ASP761 and ALA762, directly identifying GLU811 and SER814 as key hydrogen-bonding residues that stabilize ligand binding [47]. Another study demonstrated that screened RdRp inhibitors formed multiple hydrogen bonds with GLU811 and SER814 and established a salt bridge with GLU811, indicating that GLU811 not only participates in hydrogen bonding but can also engage in electrostatic interactions to stabilize the ligand [48]. Other molecular docking and dynamics simulations of SARS-CoV-2 RdRp have consistently observed GLU811 and SER814 as common contact residues for nucleoside triphosphates or designed antagonists, supporting their roles in substrate positioning and maintenance of the catalytic-site geometry [49]. In our docking model, a carbon–hydrogen bond interaction involving G15 was observed within the RdRp-Molecule_36 complex. Although G15 is located in the nidovirus RdRp-associated nucleotidyltransferase (NiRAN) domain rather than the catalytic palm subdomain, this residue may help stabilize the local conformation through weak C–H···O contacts, thereby supporting ligand anchoring and maintaining overall RdRp structural integrity [50,51]. This interaction may indicate cross-domain stabilization bridging the NiRAN and palm subdomains, suggesting a structural coupling between catalytic and regulatory regions that contributes to RdRp conformational stability. In addition to the shared hydrogen-bonding interactions at GLU811 and SER814, both ligands exhibited distinct secondary interaction networks that contribute to the overall stability of the RdRp-ligand complex.

For Molnupiravir, four categories of interactions were identified. First, conventional hydrogen bonds were formed with TRP617, ASP761, ALA762, GLU811, and SER814, residues situated within the palm subdomain that are essential for template stabilization and metal-ion coordination during RNA chain elongation [26,47,52,53]. Second, carbon–hydrogen bonds with LYS798 and G15 further reinforced the ligand’s anchoring by adding weak electrostatic and hydrophobic stabilization across both the palm and NiRAN domains [26,50,51]. Third, a Pi-Anion interaction was observed, indicating an electrostatic attraction between the aromatic ring of the ligand’s base moiety and a nearby negatively charged residue, which helps orient the ligand within the active site pocket [54,55,56,57]. Finally, an unfavorable donor–donor interaction was noted at ALA762, suggesting a potential electrostatic repulsion between overlapping hydrogen donors; this may reflect transient or non-productive binding geometries inherent to the nucleoside analog scaffold of Molnupiravir [26,47,58,59]. In contrast, Molecule_36 displayed five types of interactions. It retained the conventional hydrogen bonds with GLU811 and SER814, preserving the key anchoring contacts characteristic of nucleoside-like ligands [47]. The molecule also formed carbon–hydrogen bonds with TRP617, ASP761, and G15, extending the interaction network toward both catalytic and structural domains [26,51]. A Pi-Alkyl interaction with TRP800 and an Alkyl contact with LYS798 introduced additional hydrophobic stabilization, which likely enhances conformational rigidity and reduces solvent exposure of the ligand [26,60]. Moreover, a localized electrostatic repulsion between negatively charged carboxylate groups near GLU811 was detected in our docking model, which may transiently arise when both ligand and residue possess proximal negative centers [47]. Such negative–negative contacts are classified as unfavorable electrostatic interactions that can transiently destabilize the complex but are frequently observed in dynamic docking ensembles [58]. Nevertheless, this type of local charge repulsion can also act as a subtle electrostatic steering or fine-tuning mechanism, guiding ligands toward an energetically favorable orientation within the catalytic cavity [61]. Collectively, both Molnupiravir and Molecule_36 engage a conserved network of interactions within the palm subdomain, notably through hydrogen bonds with GLU811 and SER814, residues essential for RNA template stabilization and catalysis. These shared contacts highlight a common anchoring mechanism consistent with nucleoside-like inhibitors targeting SARS-CoV-2 RdRp. However, Molecule_36 differs from Molnupiravir in several important aspects. It extends the interaction network toward the NiRAN domain via a C–H···O contact with G15 and introduces additional hydrophobic (Pi-Alkyl and Alkyl) interactions involving TRP800 and LYS798, which may enhance binding stability and conformational rigidity. In contrast, Molnupiravir primarily relies on a denser hydrogen-bonding network but also exhibits an unfavorable donor–donor contact at ALA762, reflecting the limitations of its nucleoside analog geometry. Overall, these comparisons suggest that while both ligands share critical palm-site anchoring motifs, Molecule_36 achieves a broader and potentially more stable binding profile through cross-domain and hydrophobic stabilization, indicating a promising optimization of Molnupiravir’s pharmacophoric framework for future antiviral design.

### 3.2. Biological and Pharmacological Implications

The observed interaction profile of Molecule_36 carries several biological and pharmacological implications. By maintaining hydrogen-bond contacts with GLU811 and SER814, which are central residues to RNA template alignment and catalysis [47], the molecule is predicted to interfere directly with the elongation phase of viral RNA synthesis. Its additional hydrophobic and C–H···O contacts extending into the NiRAN domain may further stabilize the polymerase in a non-productive conformation, potentially hindering the transition between initiation and elongation cycles; such weak hydrogen-bond-like and hydrophobic interactions are known to stabilize protein–ligand complexes, and cross-domain coupling between the NiRAN and polymerase core has been shown to contribute to RdRp structural stability and catalytic regulation [45,62,63,64,65].

From a drug-design perspective, Molecule_36 combines key pharmacophoric features of Molnupiravir with improved physicochemical and ADMET properties, supporting its tractability as a small-molecule antiviral lead. The MD analysis provides additional insight into the interaction behavior of Molecule_36 and the RdRp. The reference RdRp structure (7OZV) displayed a short equilibration phase, reaching an RMSD of ~2–3 Å within the first 5 ns, after which it remained tightly stabilized around 3 Å for the remainder of the 100 ns simulation. This stable plateau is consistent with prior MD studies showing that SARS-CoV-2 RdRp maintains a rigid and well-packed polymerase core due to extensive intra-domain hydrogen bonding and conserved catalytic architecture [43,66]. The stability of the reference trajectory indicates that the system preparation and simulation parameters were appropriate and that the polymerase backbone remained structurally intact throughout the simulation. By comparison, the RdRp-Molecule_36 complexes showed a higher but still bounded RMSD profile. RMSD increased rapidly during the first 5 ns to ~7–8 Å, reflecting early relaxation and accommodation of the ligand within the binding pocket. From approximately 10 ns onward, the RMSD fluctuated within a relatively stable range of ~7–9 Å, with only a transient rise near 60–70 ns that subsequently relaxed back into the same window. This stable yet dynamic behavior is consistent with prior MD studies which confirm that RdRp complexes reach a dynamic equilibrium and maintain stability throughout the simulation trajectory while preserving a structurally conserved polymerase core [67,68]. The broader motions observed for Molecule_36 likely reflect its more extended interaction network, spanning both the palm and NiRAN domains, as suggested by the docking results. Prior MD studies of RdRp-ligand complexes have similarly reported cross-domain adjustments when inhibitors engage residues beyond the canonical palm site [67,68]. Weak C–H···O interactions with G15 and hydrophobic contacts with TRP800 may contribute to these local rearrangements, consistent with structural analyses showing that such weak hydrogen-bond-like contacts and hydrophobic interactions frequently support ligand stabilization and enable subtle induced-fit adaptations in protein–ligand complexes [63,64,69]. This behavior may also be functionally relevant, as previous RdRp studies have demonstrated that ligands promoting moderate backbone mobility can interfere with template translocation or catalytic synchronization rather than acting solely through chain termination [51,70].

The integration of deep generative modeling with cheminformatics-based filtering represents a promising strategy for rapidly exploring novel chemical spaces around validated antiviral scaffolds. These findings highlight how AI-assisted molecular generation, guided by structural insights into RdRp, may accelerate the discovery of selective inhibitors with enhanced binding stability and reduced off-target liability.

### 3.3. AI-Assisted Drug Design Framework

In recent years, AI-assisted drug discovery has reshaped early-stage antiviral research by integrating molecular representation learning, generative modeling, and structure-based evaluation within a unified workflow [71]. Strategies such as ligand-based similarity search, pharmacophore-based virtual screening, and molecular docking remain foundational [72]; however, their strength lies mainly in the repurposing of existing drugs like Remdesivir, Molnupiravir, Galidesivir, Ribavirin, Sofosbuvir, Tenofovir, and Favipiravir [73]. In contrast, AI-driven generative approaches can transcend these limitations by directly proposing novel small molecules with drug-like properties and optimized ADMET profiles, thereby addressing the need for first-in-class antiviral candidates [74]. Several machine-learning paradigms have been applied in this context, including convolutional neural networks (CNNs) and recurrent neural networks (RNNs) for feature extraction, variational autoencoders (VAEs) and generative adversarial networks (GANs) for molecular generation, and reinforcement learning (RL) for reward-guided chemical optimization [75]. For example, Beck et al. employed a deep-learning-based drug-target interaction model (MT-DTI) using CNN and Transformer architectures to predict binding affinities between FDA-approved drugs and SARS-CoV-2 proteins [76,77]. Olivecrona et al. (2017) introduced a pioneering RL framework for de novo molecular design, where a SMILES-based RNN pretrained on existing compounds was fine-tuned via policy-gradient optimization using an augmented likelihood objective to balance exploration and chemical realism; this approach enabled generation of novel, synthesizable molecules optimized for desired properties such as activity and drug-likeness, establishing a key foundation for later RL-driven generative drug-design methods [15]. Similarly, Zhavoronkov et al. demonstrated a GAN-derived deep generative framework that produced novel 3CLpro inhibitors within weeks of model training [17]. Bung et al. further applied an RNN-based deep reinforcement learning (deep RL) approach to design candidate small molecules targeting the SARS-CoV-2 3CL protease, showing conceptual similarity to the generative process employed in this study [19]. However, while Bung et al. relied on transfer learning from the ChEMBL database of drug-like compounds [19] to retrain their model, our approach utilized Molnupiravir-like chemical scaffolds from the BIOVIA ACD database as seed structures to train a mGENTRL. In addition, their compound screening relied mainly on drug-likeness rules and physicochemical property filters, whereas our workflow integrates VAEs-RL in coupling with pharmacophore-based virtual screening and structure-based docking to refine candidate selection. Building upon these advances, our study integrates a mGENTRL with cheminformatics-guided screening to enable the de novo generation and prioritization of potential SARS-CoV-2 RdRp inhibitors. Unlike models focused solely on affinity prediction or generative exploration, our workflow combines de novo molecular generation, pharmacophore-based filtering, and structure-based docking, providing an end-to-end framework for both creation and rational triage of antiviral candidates.

### 3.4. Limitations and Future Directions

In this study, there were some limitations. First, the training of the similarity search and generative model relied on the ACD database, which inherently limits the chemical-space diversity of the generated candidates. Second, Molecule_36 has not yet been tested in vitro or in vivo and should therefore be regarded as a computationally generated scaffold rather than a finalized drug-like molecule. Additional chemical refinement and experimental validation will be required to determine its stability, mechanism of action, and antiviral efficacy.

## 4. Materials and Methods

### 4.1. Study Design and Process

This study is to develop potential anti-SARS-CoV-2 compounds using a generative deep neural networks (GDNN) combined with cheminformatics [16,78,79]. The molecule formula of Molnupiravir (C_13_H_19_N_3_O_7_) is served as a raw model of the training dataset in GDNN and is shown in Supplementary Figure S1. To obtain similar chemical structures of Molnupiravir, the database of BIOVIA Available Chemicals Directory version 2020.08 (BIOVIA ACD) was screened using the method of ligand-based similarity search in the BIOVIA Discovery Studio (DS) software (version 2022) [80].

After the establishment of the training dataset, a reinforcement learning model was used to generate small molecular structures. Subsequently, pharmacophore-based virtual screening of small molecules was performed to identify similar characteristics of Molnupiravir. We used molecular docking simulation with Molnupiravir as the positive control to select compounds from pharmacophore-based virtual screening to identify potential new compounds with higher affinity with SARS-CoV-2 RdRp.

**Figure 6:**
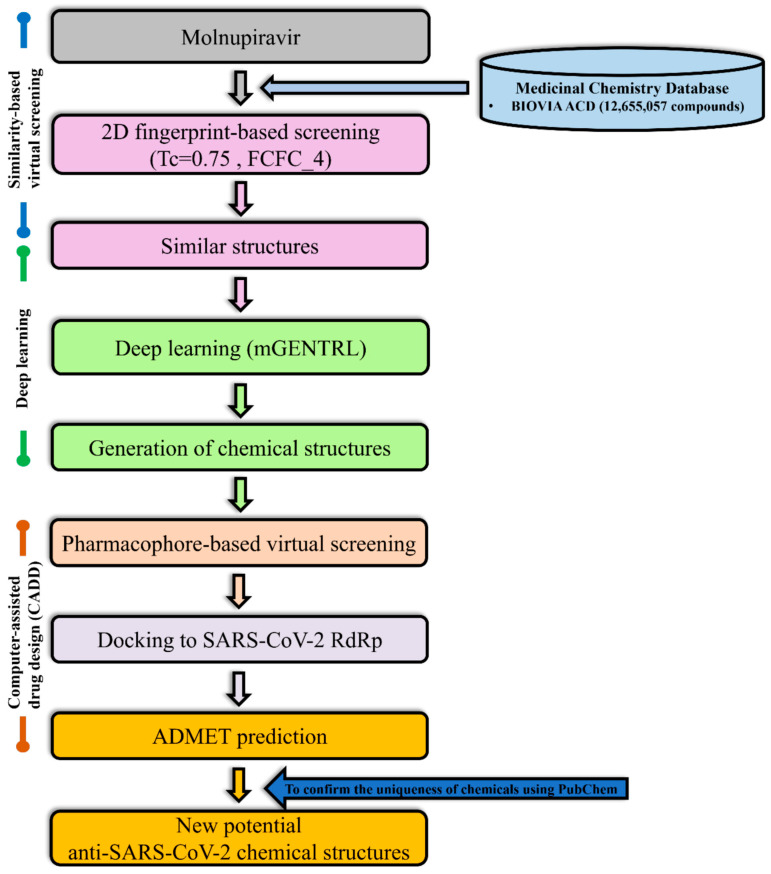
Flowchart. Tc: Tanimoto Coefficient. FCFC_4: Count vector form of FCFP4. mGENTRL: Modified generative tensorial reinforcement learning. SARS-CoV-2: Severe Acute Respiratory Syndrome Coronavirus 2. RdRp: RNA-dependent RNA polymerase. ADMET: Absorption, distribution, metabolism, excretion, and toxicity.

Finally, ADMET analysis was applied to confirm that the candidates for small molecules were suitable. Patent information was checked using PubChem [81] and Reaxys [82]. The flowchart was shown in Figure 6.

### 4.2. Preprocessing of Data and Ligand-Based Similarity Search

Molnupiravir was used as the target for preliminary screening of its similar chemical structure based on the principle that structurally similar compounds have similar medicinal chemical properties [83]. The SMILES format of Molnupiravir was from the “Canonical SMILES” field in PubChem (Compound CID: 145996610). Furthermore, the database, BIOVIA ACD contains 126,550,570 drug-like small molecules and is currently one of the most abundant commercial compound collection databases in the world, including information on unique compounds from nearly 900 suppliers [80].

The relevant conditional process for ligand preprocessing using DS software was as follows: The ligand structure was repaired and prepared by the “Prepare Ligands” function. Then, the conformation that met the conditions for oral drugs was screened out by the “Filter by Lipinski and Veber Rules” function; finally, the ligand structure was optimized by the “Full Minimization” function to obtain the structure of a small molecule with the lowest energy.

To seek ligand similarity, we used the “Find Similar Molecules by Fingerprints” function in DS whose criteria referred to Pavadai et al., 2017 [84], to screen out small molecules with structures similar to Molnupiravir. For similarity calculation, we used the Tanimoto coefficient with a minimum similarity threshold of 0.75 and the FCFC4 algorithm for similarity search [84]. Sequentially, the filtered chemical structures were used as a training dataset in GDNN.

### 4.3. Establishment of the Deep Generative Model

The deep generative model (mGENTRL) was established from the modified GENTRL model which was based on the adjustment of the reward function, using Lipinski’s rule of five as the setting of the screening conditions [85]. The preprocessed chemical database was fed into the mGENTRL model for training, with the learning rate of the model parameter set to 0.0004. Please visit GitHub (modified GENTRL model) for more detailed code content. In addition, the “valid perc” parameter in the GENTRL model was used to calculate the percentage of valid SMILES in the newly generated SMILES string to evaluate the relative performance of the created mGENTRL model [86]. All computations of training were executed on PyTorch in Python 3.6.5 version through Anaconda3-5.2.0-Windows-x86_64 version and with the help of NVIDIA GeForce RTX 3070 graphics card.

### 4.4. Pharmacophore-Based Virtual Screening

The new compounds sampled by the mGENTRL model were screened further using a pharmacophore-based method based on Molnupiravir structure to sieve new small molecule compounds with similar chemical properties to Molnupiravir. First, a Molnupiravir-based pharmacophore model was generated by the “Common Feature Pharmacophore Generation” function, and the relevant conditional process for creating the pharmacophore model using DS software was as follows: “BEST” in “Conformation Generation” was selected to ensure optimal coverage of the conformational space. “Maximum Conformations” was set to 200 and “Energy Threshold” was set to 10 kcal/mol to ensure that a maximum of 200 conformations were generated for each small molecule to characterize its conformational space, among which only those within the energy threshold of 10 kcal/mol were retained. “Features” contained the hydrogen bond acceptor (HB_ACCEPTOR), hydrogen bond donor (HB_DONOR), hydrophobicity (HYDROPHOBIC), positive ion (POS_IONIZABLE), and aromatic ring center (RING_AROMATIC), and the rest of the parameter settings remained the default values.

Subsequently, the new small molecules sampled from the mGENTRL models were used to create a proprietary 3D chemical database by the “Build 3D Database” function, and the relevant conditional process for building a 3D database using DS software was as follows: “Number of Conformations” was set to 200 and “Conformation Method” was set to “BEST” to ensure the best coverage of 200 spatial conformations for each small molecule.

Finally, the pharmacophore-based virtual screening was performed on the 3D chemical database created above through the “Search 3D Database” function, where the “Search Method” was selected as “BEST” to identify structurally novel and potential small molecule compounds.

### 4.5. Molecular Docking

To further identify potent small molecule compounds against SARS-CoV-2, a model of molecular docking was used. First, SARS-CoV-2 RdRp, the 3D electron microscopic crystal structure from PDB ID: 7OZV [87], was served as a target protein. Preprocessed tasks included the removal of water molecules and heteroatoms present in this crystal structure. The active site coordinates of SARS-CoV-2 RdRp were referred to Aftab et al., 2020 [26]. Small molecule compounds filtered by pharmacophore-based virtual screening above were fed into a model of molecular docking. We performed molecular docking simulations with “Dock Ligands (CDOCKER)” [88], with the Pose Cluster Radius set to 0.5 to ensure the greatest possible diversity of docked conformations and all other parameters kept at default values. Finally, the criteria to identify candidate small molecules based on better binding affinity is set when compared to that of Molnupiravir and 7OZV.

### 4.6. Prediction of ADMET Properties

In this study, tools such as SwissADME [89], pkCSM [90], and DS software were used to predict and analyze the ADMET properties of small molecule compounds to confirm their potential to be drugs. The “ADMET Descriptors” function of the DS software predicts numerous pharmacological properties of small molecule compounds such as Aqueous solubility, blood–brain barrier penetration (BBB), Cytochrome P4502D6 inhibition, hepatotoxicity, human intestinal absorption, and plasma protein binding for selected small molecule compounds. The “Toxicity Prediction” function can also predict carcinogenicity, mutagenicity, skin sensitization, developmental toxicity potential, Rat Oral LD50, and biodegradability [91].

### 4.7. Analysis of Molecular Dynamics Simulations

Refined protein–ligand complexes were subjected to all-atom MD simulations using the CHARMM36 force field implemented in DS (version 2024). The system was solvated in a box with TIP3P water spanning 7 Å from any solute atom and neutralized with Na^+^/Cl^−^ ions to 0.15 M ionic concentration. Periodic boundary conditions were employed in all directions. Nonbonded interactions were calculated using a 12 Å cutoff with a switching function beginning at 10 Å, and long-range electrostatics were handled with the Particle Mesh Ewald method. Bond distances involving hydrogen atoms were restricted using the SHAKE algorithm, allowing a 2 fs integration time step. Temperature was set at 310 K via Langevin dynamics (damping coefficient: 1 ps^−1^), and pressure was controlled at 1 atm using the Langevin piston method. After the standard dynamics cascade, production MD simulations were continued in NAMD without positional restraints. Trajectories were recorded every 50 ps for subsequent analyses. Two independent molecular dynamics simulations were performed, with each run extending for 100 nanoseconds (ns).

## 5. Conclusions

In summary, this study explores a workflow that combines a deep generative neural network with cheminformatics-based screening to support the rapid identification of potential small-molecule inhibitors targeting SARS-CoV-2 RdRp. Among the generated compounds, Molecule_36 showed encouraging predicted binding interactions and ADMET characteristics, and no exact-structure patent records were found in an extended public patent search. While further synthesis and biological evaluation are required, this approach provides a feasible direction and a practical framework for accelerating the early discovery of new antiviral candidates.

## Figures and Tables

**Table 1:** Comparison of potential new small molecule compounds with Molnupiravir in the prediction of ADMET properties.

Parameters			Molecule_36	Molnupiravir
Absorption	Aqueous solubility ^c^		−1.032	−0.894
	Human intestinal absorption ^a,c^		Good	Low
	Caco-2 permeability ^b^ (log cm/s)		1.082	0.531
	P-glycoprotein substrate ^a,b^		No	No
	Bioavailibility score ^a^		0.56	0.55
Distribution	VDss ^b^ (human, (log L/kg))		−0.379	0.581
	BBB permeability ^b^ (log BB)		−0.67	−1.057
	CNS permeability ^b^ (log PS)		−3.143	−3.761
Metabolism	CYP1A2 inhibitior ^a,b^		No	No
	CYP2C19 inhibitior ^a,b^		No	No
	CYP2C9 inhibitior ^a,b^		No	No
	CYP2D6 inhibitior ^a,b,c^		No	No
	CYP3A4 inhibitior ^a,b^		No	No
Excretion	Total clearance ^b^ (log ml/min/kg)		0.671	0.203
	Renal OCT2 substrate ^b^		No	No
Toxicity	Developmental Toxicity Potential ^c^		No	Yes
	Oral Rat LD_50_ ^c^		6.58	3.03
	Carcinogenicity ^c^		No	No
	Mutagenicity ^c^		No	No
	Hepatotoxicity ^b,c^		No	Yes
	Cardio-toxicity	hERG I inhibitor ^b^	No	No
		hERG II inhibitor ^b^	No	No
	Skin sensitization ^b,c^		No	No
	Biodegradability ^c^		Yes	Yes

^a^ The result of using the online service Swissadme; ^b^ the result of using the online service pkCSM; ^c^ the result of using the Discovery Studio software (version 2022).

## Data Availability

The code used in this study is openly available on GitHub at https://github.com/young19990726/mGENTRL-model (accessed on 10 October 2025). The molecular data used for model training was obtained from the BIOVIA Available Chemicals Directory (BIOVIA ACD) database, which is a commercial dataset. The authors are not permitted to redistribute the dataset.

## Supplementary Materials

The following supporting information can be downloaded at https://www.mdpi.com/article/10.3390/ijms262412017/s1.
