# Supplementary material for: Designing Novel Compound Candidates Against SARS-CoV-2 Using Generative Deep Neural Networks and Cheminformatics
Source: Int J Mol Sci. 2025 Dec 13;26(24):12017. doi: 10.3390/ijms262412017 (PMC12732431; doi:10.3390/ijms262412017)
Supplement: Supplementary file 1 [file ijms-26-12017-s001.zip › ijms-3956460-supplementary.pdf]

Supplementary document

A

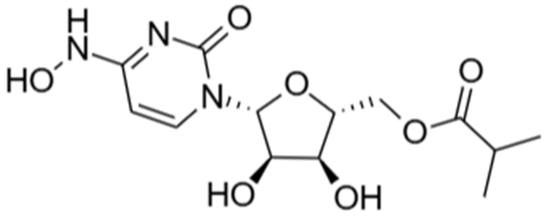

B

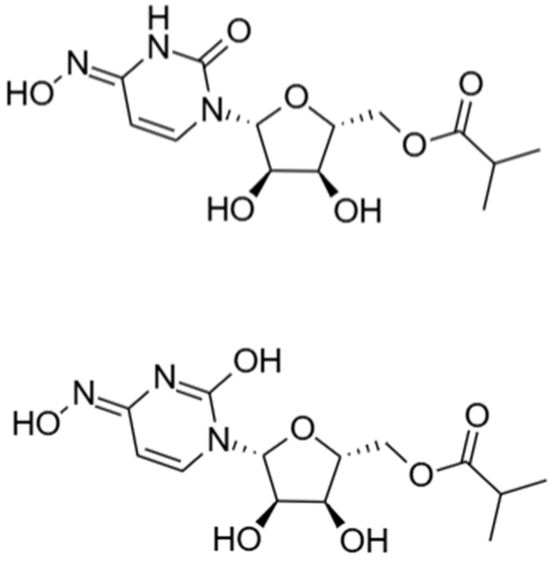

Supplementary Figure S1. Prediction of resonance structures of Molnupiravir.

A. Original structure of Molnupiravir.

B. Potential resonance structures of Molnupiravir.

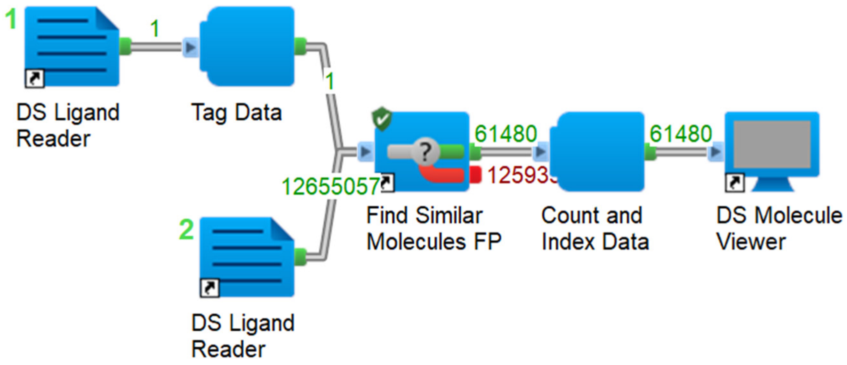

Supplementary Figure S2. Process of ligand-based similarity search.
Ligand-based similarity search based on matching between Molnupiravir and structures from the ACD database was conducted using DS software version 2022.

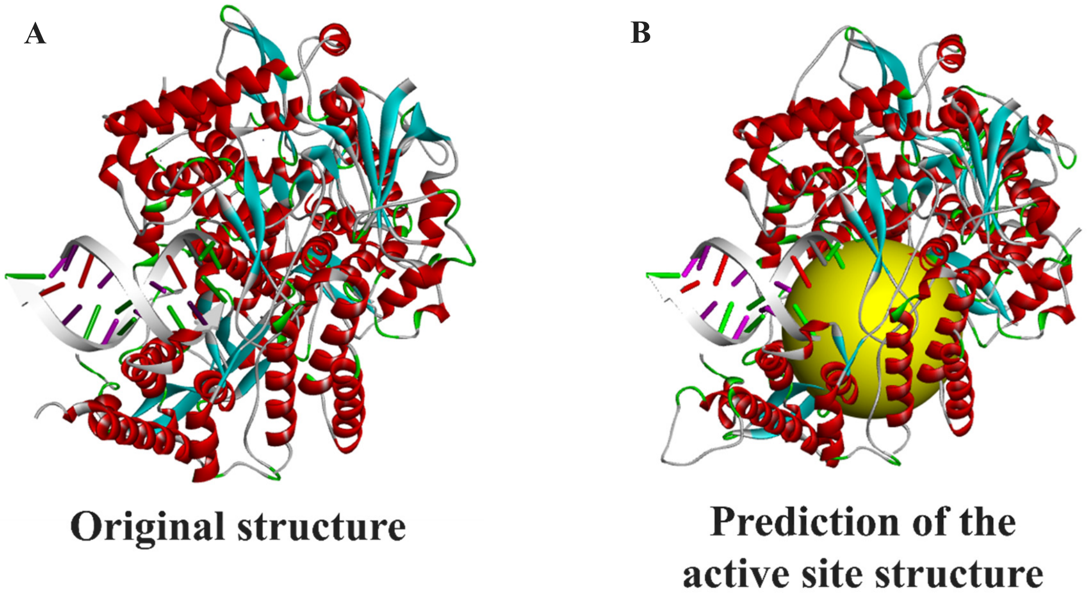

Supplementary Figure S3. SARS-CoV-2 RdRp protein and prediction of the active site (yellow sphere). A. Structure of SARS-CoV-2 RdRp protein was from PDB (ID: 7OZV). B. Prediction of the active site (yellow sphere) in preprocessed SARS-CoV-2 RdRp protein was conducted by using DS software. The coordinates and radius of the active sites were 98.562232 at the X-axis, 89.153913 at the Y-axis, 97.502913 at the Z-axis, and a radius of 15.844569, respectively.

ID	Canonical SMILES	Molecular Weight	CDOCKER_ INTERACTION_ENERGY (-kcal/mol)
**Molnupiravir**	CC(C)C(=O)OCC1C(C(C(O1)N2C=CC(=NC2=O)NO)O)O	329.31	40.5728
**Molecule 1**	CCOC(=O)/C=C(/CC[C@@H](C(=O)NC)N)\[O-]	229.252975	57.4167224
**Molecule 2**	CN(CC(=O)OCC)CC(=O)N[C@@H]([C@H]([NH3+])C(=S)N)C	291.39032	55.9340406
**Molecule 3**	CCOC(=O)CSC[C@@H](C(=O)NC[C@@H](C[NH3+])C)N	278.391571	55.8680118
**Molecule 4**	CCOC(=O)[C@@H]([NH2+]CC[C@@H](C(=O)NC(C)C)N)C	260.353149	55.6858644
**Molecule 5**	CCOC(=O)CCNC(=O)C[NH2+][C@@H](C(=O)NC)C	260.310089	55.2926295
**Molecule 6**	CCOC(=O)CNC(=O)[C@H]([NH2+][C@@H](C(=O)NC)C)C	260.310089	54.8672859
**Molecule 7**	CCOC(=O)C[C@@H](N[C@@H](C(=O)NCC(C)C)C)[NH3+]	260.353149	54.8577308
**Molecule 8**	CCOC(=O)[C@H](NC[C@@H](C(=O)N[C@H]([NH3+])C)C)C	246.326553	54.498203
**Molecule 9**	CCSC[C@@H](C(=O)N[C@@H](CC(=O)NC)C)[NH3+]	248.365585	54.2888649
**Molecule 10**	CCOC(=O)CC[C@@H](C[N@@H+](CC(=O)NC)C)N	246.326553	54.2648874
**Molecule 11**	C[C@@H]([NH2+]CC(=O)OCC)CC(=O)N[C@@H](C(=O)N)C	260.310089	54.1420374
**Molecule 12**	CN(CC(=O)OCC)CC(=O)N[C@@H]([C@H]([NH3+])C(=N)S)C	291.39032	54.1182754
**Molecule 13**	CCOC(=O)CN[C@@H](C(=O)N[C@@H](/C(=[NH+]\C)/N)C)C	259.325317	53.9230995
**Molecule 14**	CCOC(=O)C[C@@H](C(=O)N[C@@H](C(=O)N)CC)[NH3+]	246.283493	53.8842595
**Molecule 15**	CSC[C@@H](C(=O)N[C@@H]([C@@H](C(=O)NC)[NH3+])C)C	248.365585	53.7372075

ID	Canonical SMILES	Molecular Weight	CDOCKER_ INTERACTION_ENERGY (-kcal/mol)
**Molecule 16**	CCSC[C@@H](C(=O)N[C@@H]([C@@H](C(=O)NC)C)C)[NH3+]	262.39215	53.7057495
**Molecule 17**	CCOC(=O)C[NH2+]CSC[C@@H](C(=O)NC(C)C)N	278.392	52.948
**Molecule 18**	CCNC(=O)[C@H]([NH2+]CCCC(=O)OCC)N	232.29997	52.9359442
**Molecule 19**	CCSC[C@@H](C(=O)NC[C@@H](C(=O)NCC)C)[NH3+]	262.39215	52.5424038
**Molecule 20**	CCSC[C@@H](C(=O)NC[C@@H](C(=O)NC)C)[NH3+]	248.36558	52.520704
**Molecule 21**	CCSC[C@@H](C(=O)N[C@@H](C[C@@H](C(=O)NC)C)C)[NH3+]	276.41873	52.0302557
**Molecule 22**	CCOC(=O)/C=C(/CC[C@@H](C(=O)NC)[NH3+])\O	231.26886	50.9643695
**Molecule 23**	C[C@H](C(=N)S)NC(=O)C[C@@H]([NH3+])C(=O)OCC	248.32252	49.4198297
**Molecule 24**	[O-]C(=O)N[C@H](CS[C@H](C(=O)OCC)N)C	235.28070	47.6548799
**Molecule 25**	CCOC(=O)CC(=O)NC[C@@H](NC(=O)[O-])C	231.2258	47.2310574
**Molecule 26**	CCSC[C@@H](C(=O)[O-])NC(=O)N(CC(=O)NCC)C	290.35919	46.1654685
**Molecule 27**	[O-]C(=O)N[C@H]([C@H](NCC(=O)OCC)C)C	217.24227	45.6586102
**Molecule 28**	CC[C@@H](C(=O)NCCC[C@@H](C(=O)NC)N)O	231.29203	44.3003149
**Molecule 29**	CCOC(=O)CCNC(=O)CN[C@@H](C(=O)NC)C	259.30215	42.8032313
**Molecule 30**	[O-]C(=O)N[C@H](CN[C@@H](C(=O)OCC)O)C	219.21510	42.3235899
**Molecule 31**	CCOC(=O)CCCNC(=O)[C@@H](NC(=O)NC)C	259.30215	42.1549319
**Molecule 32**	CCOC(=O)CNC(=O)[C@H](N[C@@H](C(=O)NC)C)C	259.30215	42.0820871

ID	Canonical SMILES	Molecular Weight	CDOCKER_ INTERACTION_ENERGY (-kcal/mol)
**Molecule 33**	CCOC(=O)[C@H](NC[C@@H](NC(=O)[C@H](N)C)C)N	246.30668	41.8943917
**Molecule 34**	CCOC(=O)CN(C(=O)NCCC(=O)NC(C)C)C	273.32870	41.6996826
**Molecule 35**	CCNC(=O)[C@H](CCSCC(=O)OCC)N	248.34240	41.6865078
**Molecule 36**	CCCNC(=O)[C@@H](NC(=O)[CH-]C(=O)OCC)C	243.27955	41.0317729
**Molecule 37**	CCOC(=O)CN(C[C@@H](NC(=O)[C@@H](N)C)C)C	245.31861	40.9210278
**Molecule 38**	CCOC(=O)CN(C[C@@H](NC(=O)CN)C)C	231.29203	40.7263223

Supplementary Table S1 Compounds screened by molecular docking simulation.

Name	Solubility_Level	BBB_Level	CYP2D6#Prediction	Hepatotoxic#Prediction	Absorption_Level	PPB#Prediction
Molecule_36	4	3	FALSE	FALSE	0	FALSE
Molnupiravir	4	4	FALSE	TRUE	2	FALSE

Supplementary Table S2. Prediction of ADMET results using DS software version 2022.

The Solubility_Level represents extremely low at level 0, very low at level 1, low at level 2, good at level 3, optimal at level 4, and too soluble at level 5, respectively. The BBB_Level represents very high at level 0, high at level 1, medium at level 2, low at level 3, and undefined at level 4, respectively. The Absorption_Level represents good absorption at level 0, moderate absorption at level 1, low absorption at level 2, and very low absorption at level 3, respectively.

Model Name	Molecule_36	Molnupiravir
**Physicochemical Properties**		
Formula	C11H19N2O4-	C13H19N3O7
MW	243.28	329.31
#Heavy atoms	17	23
#Aromatic heavy atoms	0	6
Fraction Csp3	0.73	0.62
#Rotatable bonds	10	6
#H-bond acceptors	4	8
#H-bond donors	2	4
Molar Refractivity	61.77	76.02
TPSA	84.5	143.14
**Lipophilicity**		
iLOGP	1.95	1.53
XLOGP3	0.57	-1.34
WLOGP	-0.22	-1.65
MLOGP	0.11	-1.15
Silicos-IT Log P	0.84	-1.82
Consensus Log P	0.65	-0.89
**Water Solubility**		
ESOL Log S	-1.05	-0.83
ESOL Solubility (mg/ml)	2.18E+01	4.82E+01
ESOL Solubility (mol/l)	8.97E-02	1.46E-01
ESOL Class	Very soluble	Very soluble
Ali Log S	-1.92	-1.17
Ali Solubility (mg/ml)	2.94E+00	2.24E+01
Ali Solubility (mol/l)	1.21E-02	6.81E-02
Ali Class	Very soluble	Very soluble
Silicos-IT LogSw	-2.41	0.12
Silicos-IT Solubility (mg/ml)	9.55E-01	4.34E+02
Silicos-IT Solubility (mol/l)	3.93E-03	1.32E+00
Silicos-IT class	Soluble	Soluble
**Pharmacokinetics**		
GI absorption	High	Low
BBB permeant	No	No
P-gp substrate	No	No
CYP1A2 inhibitor	No	No
CYP2C19 inhibitor	No	No

Model Name	Molecule_36	Molnupiravir
CYP2C9 inhibitor	No	No
CYP2D6 inhibitor	No	No
CYP3A4 inhibitor	No	No
log Kp (skin permeation) (cm/s)	-7.38	-9.26
**Druglikeness**		
Lipinski #violations	0	0
Ghose #violations	0	1
Veber #violations	0	1
Egan #violations	0	1
Muegge #violations	0	0
Bioavailability Score	0.56	0.55
**Medicinal Chemistry**		
PAINS #alerts	0	0
Brenk #alerts	2	1
Leadlikeness #violations	2	0
Synthetic Accessibility	2.5	4.49

Supplementary Table S3. Prediction of ADMET results using SwissADME.

Property	Model Name	Molecule_36 Predicted Value	Molnupiravir Predicted Value	Unit
Absorption	Water solubility	-1.226	-2.162	Numeric (log mol/L)
Absorption	Caco2 permeability	1.082	0.531	Numeric (log Papp in 10^{-6} cm/s)
Absorption	Intestinal absorption (human)	84.52	53.464	Numeric (% Absorbed)
Absorption	Skin Permeability	-3.552	-2.735	Numeric (log Kp)
Absorption	P-glycoprotein substrate	No	No	Categorical (Yes/No)
Absorption	P-glycoprotein I inhibitor	No	No	Categorical (Yes/No)
Absorption	P-glycoprotein II inhibitor	No	No	Categorical (Yes/No)
Distribution	VDss (human)	-0.379	0.581	Numeric (log L/kg)
Distribution	Fraction unbound (human)	0.724	0.67	Numeric (Fu)
Distribution	BBB permeability	-0.67	-1.057	Numeric (log BB)
Distribution	CNS permeability	-3.143	-3.761	Numeric (log PS)
Metabolism	CYP2D6 substrate	No	No	Categorical (Yes/No)
Metabolism	CYP3A4 substrate	No	No	Categorical (Yes/No)
Metabolism	CYP1A2 inhibitior	No	No	Categorical (Yes/No)
Metabolism	CYP2C19 inhibitior	No	No	Categorical (Yes/No)
Metabolism	CYP2C9 inhibitior	No	No	Categorical (Yes/No)
Metabolism	CYP2D6 inhibitior	No	No	Categorical (Yes/No)
Metabolism	CYP3A4 inhibitior	No	No	Categorical (Yes/No)

Property	Model Name	Molecule 36 Predicted Value	Molnupiravir Predicted Value	Unit
Excretion	Total Clearance	0.671	0.203	Numeric (log ml/min/kg)
Excretion	Renal OCT2 substrate	No	No	Categorical (Yes/No)
Toxicity	AMES toxicity	Yes	No	Categorical (Yes/No)
Toxicity	Max. tolerated dose (human)	0.974	0.28	Numeric (log mg/kg/day)
Toxicity	hERG I inhibitor	No	No	Categorical (Yes/No)
Toxicity	hERG II inhibitor	No	No	Categorical (Yes/No)
Toxicity	Oral Rat Acute Toxicity (LD50)	2.165	2.158	Numeric (mol/kg)
Toxicity	Oral Rat Chronic Toxicity (LOAEL)	2.036	2.832	Numeric (log mg/kg_bw/day)
Toxicity	Hepatotoxicity	No	Yes	Categorical (Yes/No)
Toxicity	Skin Sensitisation	No	No	Categorical (Yes/No)
Toxicity	*T.Pyriformis* toxicity	-0.511	0.285	Numeric (log ug/L)
Toxicity	Minnow toxicity	2.729	3.386	Numeric (log mM)

Supplementary Table S4. Prediction of ADMET results using pkCSM.

Molecule_36

TOPKAT_Aerobic_Biodegradability

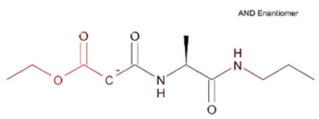

$C_{11}H_{19}N_2O_4$
Molecular Weight: 243.27956
ALogP: 0.663
Rotatable Bonds: 8
Acceptors: 4
Donors: 2

Structural Similar Compounds

Name	1,2-Propanediol,_3-(2-methoxyphenoxy)-,_1-carbamate	Butanedioic_acid,_2,3-dibromo-	Cycloheximide
Structure	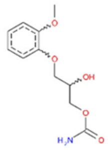	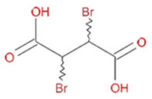	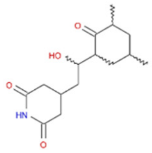
Actual Endpoint	Non-Degradable	Non-Degradable	Non-Degradable
Predicted Endpoint	Non-Degradable	Non-Degradable	Non-Degradable
Distance	0.580	0.637	0.645
Reference	Environmental Toxicology & Chemistry 18(9), 1763-1768, 1999.	Environmental Toxicology & Chemistry 18(9), 1763-1768, 1999.	Environmental Toxicology & Chemistry 18(9), 1763-1768, 1999.

Model Prediction

Prediction: Degradable
Bayesian Score: 8.61
Mahalanobis Distance: 11.3
Mahalanobis Distance p-value: 0.00559

Prediction: Positive if the Bayesian score is above the estimated best cutoff value from minimizing the false positive and false negative rate.

Bayesian Score: The standard Laplacian-modified Bayesian score.

Mahalanobis Distance: The Mahalanobis distance (MD) is the distance to the center of the training data. The larger the MD, the less trustworthy the prediction.

Mahalanobis Distance p-value: The p-value gives the fraction of training data with an MD greater than or equal to the one for the given sample, assuming normally distributed data. The smaller the p-value, the less trustworthy the prediciton. For highly non-normal X properties (e.g., fingerprints), the MD p-value is wildly inaccurate.

Model Applicability

Unknown features are fingerprint features in the query molecule, but not found or appearing too infrequently in the training set.

1. All properties and OPS components are within expected ranges.
2. Unknown SCFP_2 feature: -1946889102: [*]N[C@@H](C)C(=[*])[*]

Feature Contribution

Top features for positive contribution

Fingerprint	Bit/Smiles	Feature Structure	Score	Degradable in training set
SCFP_12	1586247563	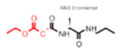 [*][CH-]C(=O)OCC	0.795	12 out of 12
SCFP_12	1132907712	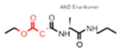 [*][CH-]C(=O)O[*]	0.791	20 out of 21
SCFP_12	276223760	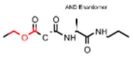 [*]COC(=[*])[*]	0.695	62 out of 75

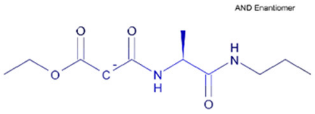

$C_{11}H_{19}N_2O_4$
Molecular Weight: 243.27956
ALogP: 0.663
Rotatable Bonds: 8
Acceptors: 4
Donors: 2

Model Prediction

Prediction: Non-Mutagen
Bayesian Score: -10.1
Mahalanobis Distance: 13.3
Mahalanobis Distance p-value: 6.71e-07

Prediction: Positive if the Bayesian score is above the estimated best cutoff value from minimizing the false positive and false negative rate.

Bayesian Score: The standard Laplacian-modified Bayesian score.

Mahalanobis Distance: The Mahalanobis distance (MD) is the distance to the center of the training data. The larger the MD, the less trustworthy the prediction.

Mahalanobis Distance p-value: The p-value gives the fraction of training data with an MD greater than or equal to the one for the given sample, assuming normally distributed data. The smaller the p-value, the less trustworthy the prediciton. For highly non-normal X properties (e.g., fingerprints), the MD p-value is wildly inaccurate.

Structural Similar Compounds

Name	58337-49-8	N-BUTYL-N-(2;4-DIHYDROXYBUTYL)NITROSAMINE	77109-49-0
Structure	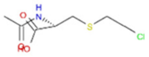	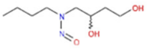	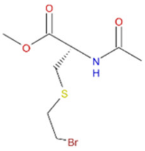
Actual Endpoint	Mutagen	Mutagen	Mutagen
Predicted Endpoint	Non-Mutagen	Mutagen	Mutagen
Distance	0.542	0.551	0.569
Reference	Kazius et. al., J. Med. Chem. (2005) 48, 312-320	EMIC	Kazius et. al., J. Med. Chem. (2005) 48, 312-320

Model Applicability

Unknown features are fingerprint features in the query molecule, but not found or appearing too infrequently in the training set.

1. All properties and OPS components are within expected ranges.

Feature Contribution

Top Features for negative contribution

Fingerprint	Bit/Smiles	Feature Structure	Score	Mutagen in training set
SCFP_12	-21815804	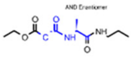 AND Enantiomer; [*][CH-]C(=O)N[C@@H](C)C(=[*])[*]	-1.49	0 out of 6
SCFP_12	-1805216672	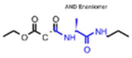 AND Enantiomer; [*]NC(=O)[C@H](C)NC(=[*])[*]	-0.762	0 out of 2
SCFP_12	-1946889102	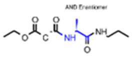 AND Enantiomer; [*]N[C@@H](C)C(=[*])[*]	-0.69	25 out of 89

Molecule_36

TOPKAT_Developmental_Toxicity_Potential

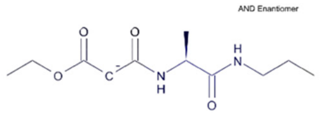

$C_{11}H_{19}N_2O_4$
Molecular Weight: 243.27956
ALogP: 0.663
Rotatable Bonds: 8
Acceptors: 4
Donors: 2

Model Prediction

Prediction: Non-Toxic
Bayesian Score: -3.37
Mahalanobis Distance: 9.39
Mahalanobis Distance p-value: 0.0851

Prediction: Positive if the Bayesian score is above the estimated best cutoff value from minimizing the false positive and false negative rate.

Bayesian Score: The standard Laplacian-modified Bayesian score.

Mahalanobis Distance: The Mahalanobis distance (MD) is the distance to the center of the training data. The larger the MD, the less trustworthy the prediction.

Mahalanobis Distance p-value: The p-value gives the fraction of training data with an MD greater than or equal to the one for the given sample, assuming normally distributed data. The smaller the p-value, the less trustworthy the prediciton. For highly non-normal X properties (e.g., fingerprints), the MD p-value is wildly inaccurate.

Structural Similar Compounds

Name	Atenolol	Bufexamac	Befunolol .HCl (Free base form)
Structure	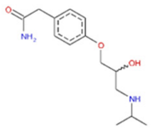	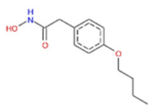	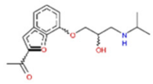
Actual Endpoint	Toxic	Toxic	Non-Toxic
Predicted Endpoint	Toxic	Toxic	Non-Toxic
Distance	0.610	0.678	0.692
Reference	Preclin Rep Cent Inst Exp Anim 6(3):253-258; 1980	Arzneimittelforschung 20(4):565-9; 1970	Kiso to Rinsho 13:3678-3714; 1979

Model Applicability

Unknown features are fingerprint features in the query molecule, but not found or appearing too infrequently in the training set.

1. All properties and OPS components are within expected ranges.

Feature Contribution

Top features for positive contribution

Fingerprint	Bit/Smiles	Feature Structure	Score	Toxic in training set
SCFP_6	9	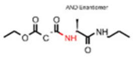 [*]N[*]	0.0928	45 out of 78
SCFP_6	-1272798659	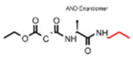 [*]CCC	0.0708	44 out of 78
SCFP_6	-711686199	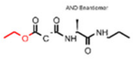 [*]OCC	0.062	33 out of 59

Top Features for negative contribution

Fingerprint	Bit/Smiles	Feature Structure	Score	Toxic in training set
SCFP_6	2005026407	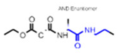 [*]CCNC(=O)C([*])[*]	-0.718	0 out of 2
SCFP_6	-1946889102	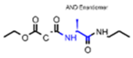 [*]N[C@@H](C)C(=[*])[*]	-0.512	4 out of 14
SCFP_6	1586247563	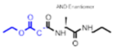 [*][CH-]C(=O)OCC	-0.438	1 out of 4

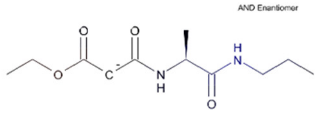

$C_{11}H_{19}N_2O_4$
Molecular Weight: 243.27956
ALogP: 0.663
Rotatable Bonds: 8
Acceptors: 4
Donors: 2

Model Prediction

Prediction: Non-Carcinogen
Bayesian Score: -3.04
Mahalanobis Distance: 7.96
Mahalanobis Distance p-value: 0.376

Prediction: Positive if the Bayesian score is above the estimated best cutoff value from minimizing the false positive and false negative rate.

Bayesian Score: The standard Laplacian-modified Bayesian score.

Mahalanobis Distance: The Mahalanobis distance (MD) is the distance to the center of the training data. The larger the MD, the less trustworthy the prediction.

Mahalanobis Distance p-value: The p-value gives the fraction of training data with an MD greater than or equal to the one for the given sample, assuming normally distributed data. The smaller the p-value, the less trustworthy the prediciton. For highly non-normal X properties (e.g., fingerprints), the MD p-value is wildly inaccurate.

Structural Similar Compounds

Name	11-Aminoundecanoic Acid	HC Blue 1	HC Blue no. 1
Structure	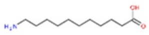	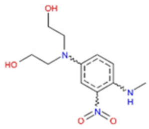	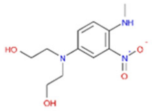
Actual Endpoint	Non-Carcinogen	Carcinogen	Carcinogen
Predicted Endpoint	Non-Carcinogen	Carcinogen	Carcinogen
Distance	0.672	0.691	0.691
Reference	NTP/TR-216	NTP/TR-271	NTP271

Model Applicability

Unknown features are fingerprint features in the query molecule, but not found or appearing too infrequently in the training set.

1. All properties and OPS components are within expected ranges.
2. Unknown ECFP_2 feature: -69385176: [*][CH-][*]
3. Unknown ECFP_2 feature: -1684029179: [*][CH-]C(=O)O[*]
4. Unknown ECFP_2 feature: -1723988332: [*][CH-]C(=O)N[*]
5. Unknown ECFP_2 feature: 975766354: [*]C([*])NC(=[*])[*]
6. Unknown ECFP_2 feature: -869848110: [*]N[C@@H](C)C(=[*])[*]
7. Unknown ECFP_2 feature: -730460647: [*]C(=[*])[CH-]C(=[*])[*]

Feature Contribution

Top features for positive contribution

Fingerprint	Bit/Smiles	Feature Structure	Score	Carcinogen in training set
ECFP_8	-1249283963	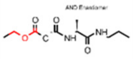 [*]COC(=[*])[*]	0.374	8 out of 14
ECFP_8	-1793471910	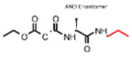 [*]CCC	0.261	8 out of 16
ECFP_8	1887306650	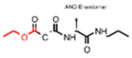 [*]C(=[*])OCC	0.216	3 out of 6

Top Features for negative contribution

Fingerprint	Bit/Smiles	Feature Structure	Score	Carcinogen in training set
ECFP_8	-649348348	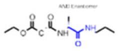 [*]NC(=O)C([*])[*]	-0.748	0 out of 3
ECFP_8	-1791034651	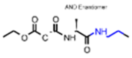 [*]CCN[*]	-0.586	1 out of 7
ECFP_8	-1897341097	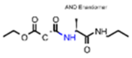 [*]N[*]	-0.555	10 out of 49

Molecule_36

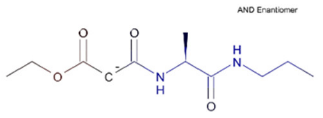

$C_{11}H_{19}N_2O_4$
Molecular Weight: 243.27956
ALogP: 0.663
Rotatable Bonds: 8
Acceptors: 4
Donors: 2

Model Prediction

Prediction: Non-Carcinogen
Bayesian Score: -6.55
Mahalanobis Distance: 9.85
Mahalanobis Distance p-value: 0.00837

Prediction: Positive if the Bayesian score is above the estimated best cutoff value from minimizing the false positive and false negative rate.

Bayesian Score: The standard Laplacian-modified Bayesian score.

Mahalanobis Distance: The Mahalanobis distance (MD) is the distance to the center of the training data. The larger the MD, the less trustworthy the prediction.

Mahalanobis Distance p-value: The p-value gives the fraction of training data with an MD greater than or equal to the one for the given sample, assuming normally distributed data. The smaller the p-value, the less trustworthy the prediciton. For highly non-normal X properties (e.g., fingerprints), the MD p-value is wildly inaccurate.

Structural Similar Compounds

Name	HC BLUE NO.1	HC Blue no. 1	PROBENECID
Structure	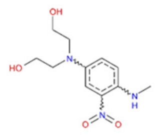	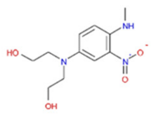	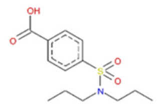
Actual Endpoint	Carcinogen	Carcinogen	Non-Carcinogen
Predicted Endpoint	Carcinogen	Carcinogen	Non-Carcinogen
Distance	0.672	0.672	0.672
Reference	NTP/TR-271	NTP271	NTP/TR-395

Model Applicability

Unknown features are fingerprint features in the query molecule, but not found or appearing too infrequently in the training set.

1. All properties and OPS components are within expected ranges.

Feature Contribution

Top features for positive contribution

Fingerprint	Bit/Smiles	Feature Structure	Score	Carcinogen in training set
SCFP_12	1132907712	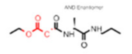 [*][CH-]C(=O)O[*]	0.476	3 out of 4
SCFP_12	276223760	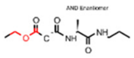 [*]COC(=[*])[*]	0.271	12 out of 24
SCFP_12	-711686199	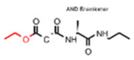 [*]OCC	0.243	23 out of 48

Top Features for negative contribution

Fingerprint	Bit/Smiles	Feature Structure	Score	Carcinogen in training set
SCFP_12	-587569116	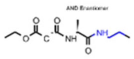 [*]CCN[*]	-0.91	0 out of 4
SCFP_12	-1946889102	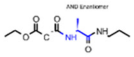 [*]N[C@@H](C)C(=[*])[*]	-0.91	0 out of 4
SCFP_12	-111024397	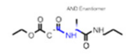 [*]C([*])NC(=[*])[*]	-0.784	2 out of 15

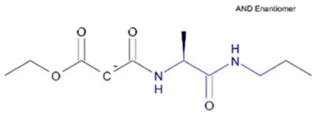

$C_{11}H_{19}N_2O_4$
Molecular Weight: 243.27956
ALogP: 0.663
Rotatable Bonds: 8
Acceptors: 4
Donors: 2

Model Prediction

Prediction: Non-Carcinogen
Bayesian Score: -5.18
Mahalanobis Distance: 9.31
Mahalanobis Distance p-value: 0.0166

Prediction: Positive if the Bayesian score is above the estimated best cutoff value from minimizing the false positive and false negative rate.

Bayesian Score: The standard Laplacian-modified Bayesian score.

Mahalanobis Distance: The Mahalanobis distance (MD) is the distance to the center of the training data. The larger the MD, the less trustworthy the prediction.

Mahalanobis Distance p-value: The p-value gives the fraction of training data with an MD greater than or equal to the one for the given sample, assuming normally distributed data. The smaller the p-value, the less trustworthy the prediciton. For highly non-normal X properties (e.g., fingerprints), the MD p-value is wildly inaccurate.

Structural Similar Compounds

Name	11-AMINOUNDECANOIC ACID	HC BLUE NO.1	HC Blue no. 1
Structure	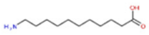	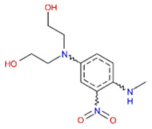	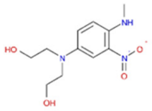
Actual Endpoint	Non-Carcinogen	Carcinogen	Carcinogen
Predicted Endpoint	Non-Carcinogen	Carcinogen	Carcinogen
Distance	0.652	0.662	0.669
Reference	TR-216	TR-271	NTP271

Model Applicability

Unknown features are fingerprint features in the query molecule, but not found or appearing too infrequently in the training set.

1. All properties and OPS components are within expected ranges.
2. Unknown FCFP_2 feature: 4: [*][CH-][*]
3. Unknown FCFP_2 feature: -2110275491: [*][CH-]C(=O)O[*]
4. Unknown FCFP_2 feature: -2110215909: [*][CH-]C(=O)N[*]
5. Unknown FCFP_2 feature: -756467783: [*]C(=[*])[CH-]C(=[*])[*]

Feature Contribution

Top features for positive contribution

Fingerprint	Bit/Smiles	Feature Structure	Score	Carcinogen in training set
FCFP_12	1	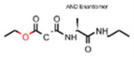 [*]O[*]	0.121	91 out of 193
FCFP_12	-1272768868	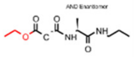 [*]OCC	0.0653	21 out of 47
FCFP_12	0	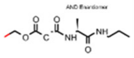 [*]C	0.0577	111 out of 251

Top Features for negative contribution

Fingerprint	Bit/Smiles	Feature Structure	Score	Carcinogen in training set
FCFP_12	159404153	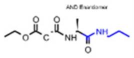 [*]C(=[*])NCCC	-0.812	0 out of 3
FCFP_12	-1043250487	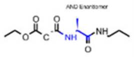 [*]N[C@@H](C)C(=[*])[*]	-0.711	4 out of 22
FCFP_12	-1272798659	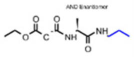 [*]CCC	-0.706	10 out of 51

Molecule_36 **TOPKAT_Rat_Male_NTP**

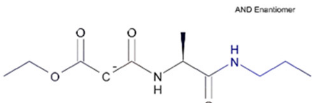

$C_{11}H_{19}N_2O_4$
Molecular Weight: 243.27956
ALogP: 0.663
Rotatable Bonds: 8
Acceptors: 4
Donors: 2

Model Prediction

Prediction: Non-Carcinogen
Bayesian Score: -4.68
Mahalanobis Distance: 7.87
Mahalanobis Distance p-value: 0.267

Prediction: Positive if the Bayesian score is above the estimated best cutoff value from minimizing the false positive and false negative rate.

Bayesian Score: The standard Laplacian-modified Bayesian score.

Mahalanobis Distance: The Mahalanobis distance (MD) is the distance to the center of the training data. The larger the MD, the less trustworthy the prediction.

Mahalanobis Distance p-value: The p-value gives the fraction of training data with an MD greater than or equal to the one for the given sample, assuming normally distributed data. The smaller the p-value, the less trustworthy the prediciton. For highly non-normal X properties (e.g., fingerprints), the MD p-value is wildly inaccurate.

Structural Similar Compounds

Name	11-Aminoundecanoic Acid	HC Blue no. 1	Probebecid
Structure	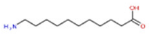	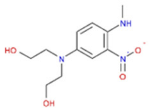	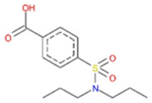
Actual Endpoint	Carcinogen	Carcinogen	Non-Carcinogen
Predicted Endpoint	Carcinogen	Carcinogen	Non-Carcinogen
Distance	0.676	0.684	0.708
Reference	NTP/TR-216	NTP271	NTP/TR-395

Model Applicability

Unknown features are fingerprint features in the query molecule, but not found or appearing too infrequently in the training set.

1. All properties and OPS components are within expected ranges.
2. Unknown ECFP_2 feature: -69385176: [*][CH-][*]
3. Unknown ECFP_2 feature: -1684029179: [*][CH-]C(=O)O[*]
4. Unknown ECFP_2 feature: -1723988332: [*][CH-]C(=O)N[*]
5. Unknown ECFP_2 feature: -869848110: [*]N[C@@H](C)C(=[*])[*]
6. Unknown ECFP_2 feature: -730460647: [*]C(=[*])[CH-]C(=[*])[*]

Feature Contribution

Top features for positive contribution

Fingerprint	Bit/Smiles	Feature Structure	Score	Carcinogen in training set
ECFP_12	975766354	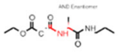 [*]C([*])NC(=[*])[*]	0.405	2 out of 2
ECFP_12	-649348348	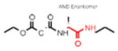 [*]NC(=O)C([*])[*]	0.288	1 out of 1
ECFP_12	-1059365320	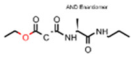 [*]O[*]	0.102	40 out of 72

Top Features for negative contribution

Fingerprint	Bit/Smiles	Feature Structure	Score	Carcinogen in training set
ECFP_12	-1791034651	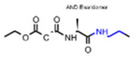 [*]CCN[*]	-1.39	0 out of 6
ECFP_12	864518973	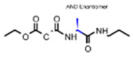 [*]C([*])C	-0.497	6 out of 21
ECFP_12	-1897341097	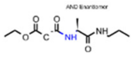 [*]N[*]	-0.429	13 out of 41

Molecule_36 **TOPKAT_Skin_Sensitization_None_vs_Sensitizer**

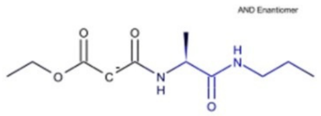

$C_{11}H_{19}N_2O_4$
Molecular Weight: 243.27956
ALogP: 0.663
Rotatable Bonds: 8
Acceptors: 4
Donors: 2

Model Prediction

Prediction: Non-Sensitizer
Bayesian Score: -4.25
Mahalanobis Distance: 8.28
Mahalanobis Distance p-value: 0.0167

Prediction: Positive if the Bayesian score is above the estimated best cutoff value from minimizing the false positive and false negative rate.

Bayesian Score: The standard Laplacian-modified Bayesian score.

Mahalanobis Distance: The Mahalanobis distance (MD) is the distance to the center of the training data. The larger the MD, the less trustworthy the prediction.

Mahalanobis Distance p-value: The p-value gives the fraction of training data with an MD greater than or equal to the one for the given sample, assuming normally distributed data. The smaller the p-value, the less trustworthy the prediciton. For highly non-normal X properties (e.g., fingerprints), the MD p-value is wildly inaccurate.

Structural Similar Compounds

Name	Propylene glycol salicylate	Ethyl lactyl pyrrolidone carboxylic acid	Guaifenesin
Structure	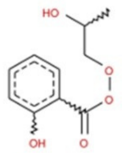	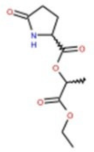	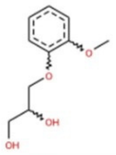
Actual Endpoint	Non-Sensitizer	Sensitizer	Non-Sensitizer
Predicted Endpoint	Non-Sensitizer	Sensitizer	Non-Sensitizer
Distance	0.626	0.642	0.659
Reference	SAR and QSAR in Env Res (1994) 2:159	SAR and QSAR in Env Res (1994) 2:159	Howard I Maibach (priv comm)

Model Applicability

Unknown features are fingerprint features in the query molecule, but not found or appearing too infrequently in the training set.

1. All properties and OPS components are within expected ranges.
2. Unknown FCFP_2 feature: 4: [*][CH-][*]
3. Unknown FCFP_2 feature: -2110275491: [*][CH-]C(=O)O[*]
4. Unknown FCFP_2 feature: -2110215909: [*][CH-]C(=O)N[*]
5. Unknown FCFP_2 feature: -756467783: [*]C(=[*])[CH-]C(=[*])[*]

Feature Contribution

Top Features for negative contribution

Fingerprint	Bit/Smiles	Feature Structure	Score	Sensitizer in training set
FCFP_12	-547731249	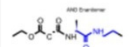 [*]CCNC(=O)C([*])[*]	-1.36	0 out of 4
FCFP_12	159404153	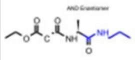 [*]C(=[*])NCCC	-0.663	1 out of 4
FCFP_12	-39956105	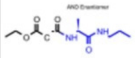 [*]N[C@@H](C)C(=O)NCC C	-0.542	0 out of 1

Molecule_36 **TOPKAT_Rat_Oral_LD50**

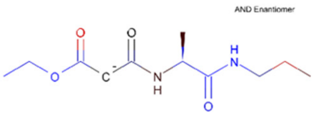

$C_{11}H_{19}N_2O_4$
Molecular Weight: 243.27956
ALogP: 0.663
Rotatable Bonds: 8
Acceptors: 4
Donors: 2

Structural Similar Compounds

Name	PHOSPHORAMIDOTHIOIC ACID; ISOPROPYL-; O-ETHYL; S-[2-(METHOXYMETHYLAMINO)-2-OXOETHYL] ESTER	CARISOPRODOL	MEPROBAMATE
Structure	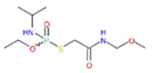	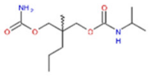	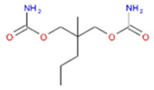
Actual Endpoint (-log C)	3.454	2.295	2.339
Predicted Endpoint (-log C)	3.53122	2.76712	2.40634
Distance	0.507	0.524	0.536
Reference	ARSIM* 20;27;66	JPETAB 127;66;59	29QHAQ -;232;74

Model Prediction

Prediction: 6.58
Unit: g/kg_body_weight
Mahalanobis Distance: 22.4
Mahalanobis Distance p-value: 5.41e-18

Mahalanobis Distance: The Mahalanobis distance (MD) is a generalization of the Euclidean distance that accounts for correlations among the X properties. It is calculated as the distance to the center of the training data. The larger the MD, the less trustworthy the prediction.

Mahalanobis Distance p-value: The p-value gives the fraction of training data with an MD greater than or equal to the one for the given sample, assuming normally distributed data. The smaller the p-value, the less trustworthy the prediciton. For highly non-normal X properties (e.g., fingerprints), the MD p-value is wildly inaccurate.

Model Applicability

Unknown features are fingerprint features in the query molecule, but not found or appearing too infrequently in the training set.

1. All properties and OPS components are within expected ranges.
2. Unknown ECFP_2 feature: -69385176: [*][CH-][*]
3. Unknown ECFP_2 feature: -1684029179: [*][CH-]C(=O)O[*]
4. Unknown ECFP_2 feature: -1723988332: [*][CH-]C(=O)N[*]
5. Unknown ECFP_2 feature: -869848110: [*]N[C@@H](C)C(=[*])[*]
6. Unknown ECFP_2 feature: -730460647: [*]C(=[*])[CH-]C(=[*])[*]
7. Unknown FCFP_6 feature: 4: [*][CH-][*]
8. Unknown FCFP_6 feature: -2110275491: [*][CH-]C(=O)O[*]
9. Unknown FCFP_6 feature: -2110215909: [*][CH-]C(=O)N[*]
10. Unknown FCFP_6 feature: -756467783: [*]C(=[*])[CH-]C(=[*])[*]

Feature Contribution

Top features for positive contribution

Fingerprint	Bit/Smiles	Feature Structure	Score
ECFP_6	-1897341097	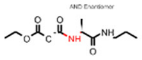 [*]N[*]	0.216
ECFP_6	-1074141656	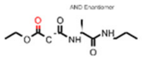 [*]=O	0.142
FCFP_6	-1272709286	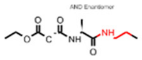 [*]CCN[*]	0.115

Top Features for negative contribution

Fingerprint	Bit/Smiles	Feature Structure	Score
ECFP_6	497523368	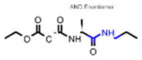 [*]CNC(=[*])[*]	-0.301
ECFP_6	1887306650	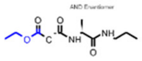 [*]C(=[*])OCC	-0.271
FCFP_6	566058135	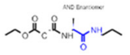 [*]NC(=O)C([*])[*]	-0.216

Supplementary Table S5. Prediction toxicity of Molecule_36 using DS software version 2022.
